# Serum High-Mobility-Group Box 1 as a Biomarker and a Therapeutic Target during Respiratory Virus Infections

**DOI:** 10.1128/mBio.00246-18

**Published:** 2018-03-13

**Authors:** Mira C. Patel, Kari Ann Shirey, Marina S. Boukhvalova, Stefanie N. Vogel, Jorge C. G. Blanco

**Affiliations:** a Sigmovir Biosystems Inc., Rockville, Maryland, USA; b Department of Microbiology and Immunology, University of Maryland, Baltimore, Maryland, USA; National Institute of Allergy and Infectious Diseases

**Keywords:** HMGB1, biomarker, influenza, respiratory syncytial virus, respiratory viruses

## Abstract

Host-derived “danger-associated molecular patterns” (DAMPs) contribute to innate immune responses and serve as markers of disease progression and severity for inflammatory and infectious diseases. There is accumulating evidence that generation of DAMPs such as oxidized phospholipids and high-mobility-group box 1 (HMGB1) during influenza virus infection leads to acute lung injury (ALI). Treatment of influenza virus-infected mice and cotton rats with the Toll-like receptor 4 (TLR4) antagonist Eritoran blocked DAMP accumulation and ameliorated influenza virus-induced ALI. However, changes in systemic HMGB1 kinetics during the course of influenza virus infection in animal models and humans have yet to establish an association of HMGB1 release with influenza virus infection. To this end, we used the cotton rat model that is permissive to nonadapted strains of influenza A and B viruses, respiratory syncytial virus (RSV), and human rhinoviruses (HRVs). Serum HMGB1 levels were measured by an enzyme-linked immunosorbent assay (ELISA) prior to infection until day 14 or 18 post-infection. Infection with either influenza A or B virus resulted in a robust increase in serum HMGB1 levels that decreased by days 14 to 18. Inoculation with the live attenuated vaccine FluMist resulted in HMGB1 levels that were significantly lower than those with infection with live influenza viruses. RSV and HRVs showed profiles of serum HMGB1 induction that were consistent with their replication and degree of lung pathology in cotton rats. We further showed that therapeutic treatment with Eritoran of cotton rats infected with influenza B virus significantly blunted serum HMGB1 levels and improved lung pathology, without inhibiting virus replication. These findings support the use of drugs that block HMGB1 to combat influenza virus-induced ALI.

## IMPORTANCE

Influenza virus is a common infectious agent causing serious seasonal epidemics, and there is urgent need to develop an alternative treatment modality for influenza virus infection. Recently, host-derived DAMPs, such as oxidized phospholipids and HMGB1, were shown to be generated during influenza virus infection and cause ALI. To establish a clear link between influenza virus infection and HMGB1 as a biomarker, we have systematically analyzed temporal patterns of serum HMGB1 release in cotton rats infected with nonadapted strains of influenza A and B viruses and compared these patterns with a live attenuated influenza vaccine and infection by other respiratory viruses. Towards development of a new therapeutic modality, we show herein that blocking serum HMGB1 levels by Eritoran improves lung pathology in influenza B virus-infected cotton rats. Our study is the first report of systemic HMGB1 as a potential biomarker of severity in respiratory virus infections and confirms that drugs that block virus-induced HMGB1 ameliorate ALI.

## INTRODUCTION

Influenza viruses cause a highly contagious respiratory infection that results in ~3 million to 5 million cases of severe illness and ~250,000 to 500,000 deaths worldwide annually (1). In the United States and most of the Northern Hemisphere, the influenza season starts in late fall and concludes in early spring, causing serious complications in young children, pregnant women, the elderly, and chronically ill patients with weakened immune systems (2).

Three types of influenza viruses infect humans: types A, B, and C (1). New antigenic variants of influenza A and B viruses emerge annually, giving rise to seasonal outbreaks. However, a new and very different influenza A virus to which humans have no prior immunity may appear due to “antigenic shift” caused by recombination of the eight-segment genome of human influenza virus with the genomes of influenza viruses of swine or avian origin. Such strains may result in catastrophic pandemics, such as the 1918 “Spanish flu” (3). Influenza A viruses are further divided into subtypes based on two proteins expressed on the virus surface: hemagglutinin (H) and neuraminidase (N). They are also further segregated into different subtypes. Current subtypes of influenza A viruses commonly prevalent in humans are influenza A H1N1 and H3N2 (4). Influenza B viruses also cause the same spectrum of symptoms and disease as caused by influenza A viruses in humans (5). However, due to the limited host range of influenza B viruses (mostly humans and marine mammals), generation of new circulating strains by recombination is limited (6). Influenza B viruses are segregated into lineages and strains. The current circulating influenza B viruses belong to one of two lineages, influenza B/Yamagata and B/Victoria (2). Surveillance data from 1994 to 2011 in the United States and Europe indicate a potentially increasing burden of influenza B in recent years (6). Influenza C virus causes mild upper respiratory tract illness and rarely lower respiratory tract complications (4).

The most common and significant strategy to combat influenza virus infection is vaccination at the start of influenza season every year. The seasonal, trivalent influenza vaccines protect against three influenza viruses, which normally include one influenza A virus of the HIN1 type, one of the H3N2 type, and one influenza B virus (usually of the Victoria lineage, B/Brisbane/60/2008) (7). However, an influenza B virus of the second lineage (Yamagata, B/Phuket/3073/2013) was included by many manufacturers in the current quadrivalent vaccines (8). It is difficult to predict the immunogenic epitopes that will be expressed annually, causing challenging issues for complete protection (9). Moreover, resistance of circulating influenza virus strains to both classes of antiviral agents, i.e., neuraminidase inhibitors and M2 channel inhibitors, poses a major concern (10). Thus, it would be highly beneficial to develop alternative therapeutic adjuncts to mitigate influenza virus-induced disease.

In 2008, Imai et al. showed that induction of acute lung injury (ALI) caused by chemical or microbial insults (infection by respiratory viruses and bacteria or exposure to their products, e.g., inactivated avian influenza virus H5N1), was mediated by NADPH oxidase-dependent production of oxidized host phospholipids, such as oxidized 1-palmitoyl-2-arachidonoyl-phosphatidylcholine (OxPAPC) (11). OxPAPC acts as a danger-associated molecular pattern (DAMP) and, in turn, stimulates Toll-like receptor 4 (TLR4), leading to a cytokine storm, which causes ALI. In support of this work, our laboratory previously showed that TLR4^−/−^ mice are protected against infection by mouse-adapted A/PR/8/34 (PR8) influenza virus and that therapeutic administration of a potent, synthetic TLR4 antagonist, Eritoran (also known as E5564), blocks PR8-induced lethality, lung pathology, clinical symptoms, and cytokine production in wild-type mice (12, 13). Subsequently, we showed that Eritoran inhibits TLR4 signaling induced by another DAMP, high-mobility-group box 1 (HMGB1) *in vitro* and HMGB1 release into serum *in vivo* and also protects mice from PR8-induced ALI (14). Like Eritoran, therapeutic administration of P5779, a highly selective HMGB1 inhibitor (15), protects mice from lethal PR8 infection (14) and ameliorates lung pathology (unpublished observations). HMGB1 is a nuclear DNA-binding protein found in eukaryotic cells that stabilizes nucleosome formation and acts as a transcription factor to regulate expression of several genes (16). HMGB1 has also been recognized as a DAMP that elicits a TLR4-dependent cytokine storm when released from necrotic cells in response to injury, infection, or other inflammatory stimuli (16, 17). HMGB1 was first implicated in endotoxicity and Gram-negative sepsis (18) and was shown to be released during severe murine influenza infection (19). HMGB1 activates TLR4 by binding to the TLR4 coreceptors CD14 and MD-2 (15, 20). Similarly, another small-molecule synthetic TLR4 antagonist, FP7, that also binds to MD-2, protects mice from PR8-induced lethality, reduces ALI and influenza virus-induced cytokine gene expression in the lungs and inhibits levels of serum HMGB1 (21). Collectively, these findings indicate that host-targeted therapeutic approaches that block TLR4-mediated signaling in response to host-derived DAMPs represent novel modalities to ameliorate influenza virus-induced ALI.

While mice have been used extensively in research to study influenza pathogenesis and host response, influenza virus must be “adapted” for mouse infection because mice lack the receptors required for infection with human isolates (22). It is possible that adaptation results in changes in the viral genome such that the course of natural infection is altered dramatically (23, 24). Moreover, most mouse laboratory strains fail to express functional Mx proteins, an interferon (IFN)-induced antiviral response that is crucial to inhibit virus replication (25). Thus, replication of influenza viruses in the lungs of mice is prolonged compared to replication in the lungs of ferrets, primates, and humans (26). Cotton rats are permissive to infection with human (A/H3N2, A/H1N1, and B), avian, and pandemic influenza virus isolates without adaptation, and influenza virus-induced clinical illness observed in cotton rats is predictive of the severity of human influenza infection (23, 27). Moreover, the cotton rat model has been validated to test the efficacy of influenza vaccines and heterosubtypic immunity to influenza virus (28, 29).

Herein, we tracked temporal changes in serum HMGB1 levels in cotton rats infected by various respiratory viruses and assessed the therapeutic potential of Eritoran to block induction of serum HMGB1 and improve influenza B virus-induced pathology. We conclude that HMGB1 may serve as a marker of disease severity in respiratory viral infections and drugs that block signaling activated in response to virus-induced DAMPs such as HMGB1 can be beneficial to mitigate influenza virus-induced disease.

## RESULTS

### Serum HMGB1 levels increase upon influenza B/Wisconsin virus infection of cotton rats.

**FIG 1:**
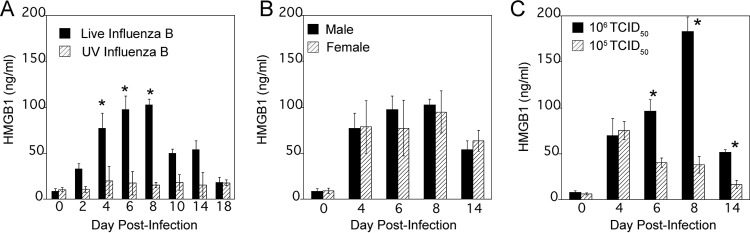
Increased serum HMGB1 levels in cotton rats infected with influenza B/Wisconsin virus. (A) Young male cotton rats were infected i.n. with 1 × 10^6^ TCID_50_/rat of influenza B/Wisconsin virus (Live Influenza B). Another group of animals similarly inoculated with UV-inactivated influenza B/Wisconsin virus (UV Influenza B) (at a dose similar to that of live virus) were used as controls. Serum samples were obtained on the indicated days p.i., and HMGB1 levels were analyzed by ELISA. Results are expressed as geometric means ± standard errors of the means (SEMs) (error bars) of the quantity of HMGB1 (in nanograms per milliliter) at each indicated time. Values for the group inoculated with live influenza virus that were significantly different (*P* < 0.05) from the values for the group inoculated with UV-inactivated influenza B virus are indicated by an asterisk. There were five cotton rats in each group for each treatment and time point. (B) Age-matched male and female cotton rats were infected i.n. with 1 × 10^6^ TCID_50_ of live influenza B virus per rat. Serum samples were obtained and analyzed for HMGB1 levels at the indicated days p.i. There were five cotton rats in each group at each time point. (C) Groups (five cotton rats in each group) were infected i.n. with either 1 × 10^5^ or 1 × 10^6^ TCID_50_ of live influenza B virus per rat, and serum HMGB1 levels were analyzed at the indicated days p.i. The values for rats infected with 1 × 10^6^ TCID_50_ that were significantly different (*P* < 0.05) from the values for rats infected with 1 × 10^5^ TCID_50_ are indicated by an asterisk.

Young male cotton rats were infected intranasally (i.n.) with 1 × 10^6^ 50% tissue culture infective doses (TCID_50_) of influenza B/Wisconsin (live influenza B) virus or inoculated i.n. with an identical amount of UV-inactivated influenza B/Wisconsin virus. Serum samples were collected from all animals before (0) and 2, 4, 6, 8, 10, 14, and 18 days post-infection (p.i.) to measure the concentration of HMGB1 in an enzyme-linked immunosorbent assay (ELISA) (Fig. 1A). Uninfected animals showed background levels of ~10 ng/ml of serum HMGB1. In response to live influenza B virus, serum HMGB1 levels gradually increased over days 2 to 4 p.i., reached peak concentrations at days 6 to 8 p.i. (~100 ng/ml), and then declined over days 10 to 14 p.i., almost reaching background levels by day 18 p.i. In contrast, inoculating animals with UV-inactivated influenza B virus failed to increase serum HMGB1 levels at any day postinoculation above 16.2 ± 1.1 ng/ml. These results indicate that induction of serum HMGB1 levels correlates with active virus replication *in vivo*. To investigate any sex imbalance for induction of serum HMGB1 concentration upon influenza B virus infection, we compared age-matched male versus female cotton rats for serum HMGB1 at 4, 6, 8, and 14 days p.i. HMGB1 levels were not significantly different in male and female cotton rats (Fig. 1B).

Next, we compared serum HMGB1 levels at different times p.i. in animals infected with either 1 × 10^6^ TCID_50_ or 1 × 10^5^ TCID_50_ of influenza B virus (Fig. 1C). Although both virus doses induced similar levels of HMGB1 at 4 days p.i., infection with 1 × 10^5^ TCID_50_ resulted in lower HMGB1 levels at later times, indicating a dose dependency.

### Serum HMGB1 induction upon infection by various strains of influenza A virus in cotton rats.

**FIG 2:**
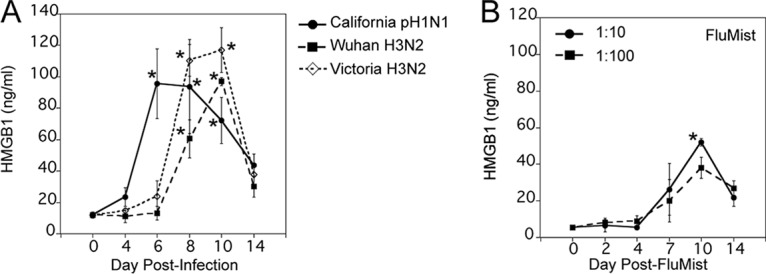
Serum HMGB1 levels increase upon infection with various strains of human influenza A viruses in cotton rats. (A) Cotton rats were infected i.n. with 1 × 10^6^ TCID_50_ of either California pH1N1 or Wuhan H3N2 virus or with 1 × 10^5^ TCID_50_ of Victoria H3N2 virus, and serum HMGB1 levels were analyzed at the indicated days p.i. Values that are significantly different (*P* < 0.05) from the value for HMGB1 on day 0 (*n* = 23) are indicated by an asterisk. (B) Groups of cotton rats (three cotton rats in each group) were i.n. inoculated with FluMist quadrivalent vaccine (MedImmune; 2015–2016 formula) either at 1:10 or 1:100 dilution (in 1× PBS), and serum HMGB1 levels were analyzed on the indicated days postvaccination. Values that were significantly different (*P* < 0.05) from the value on day 0 indicated by an asterisk.

We previously reported that influenza California pandemic H1N1 (pH1N1) virus infection induced serum HMGB1 levels in cotton rats at days 4 and 6 p.i.; however, we did not monitor the kinetics of HMGB1 induced by other influenza A strains (14). Currently, along with influenza A H1N1, H3N2 subtypes are also commonly prevalent in humans (4), and our group has previously shown that influenza A viruses with H3N2 subtypes also replicate in nose and lung tissues of cotton rats and cause significant lung inflammation (23, 27). Therefore, we next compared the response of cotton rats to three influenza A strains, California pH1N1, Wuhan H3N2, and Victoria H3N2. These three strains were prepared either in eggs (California pH1N1 and Wuhan H3N2) or Madin-Darby canine kidney (MDCK) cells (Victoria H3N2) at different times, so we used the maximum possible dose for each strain to infect groups (each group containing five animals): 1 × 10^6^ TCID_50_ for California pH1N1 and Wuhan H3N2 and 1 × 10^5^ TCID_50_ for Victoria H3N2. Serum HMGB1 levels were analyzed at 0, 4, 6, 8, 10, and 14 days p.i. As shown for influenza B virus infection (Fig. 1A), California pH1N1 virus infection induced a similar temporal pattern of HMGB1 release in serum, with a gradual increase over days 0 to 4 p.i., reaching peak concentrations at days 6 to 8 p.i., and finally declining by days 10 to 14 p.i. (Fig. 2A). Wuhan H3N2 and Victoria H3N2 strains induced a delayed temporal pattern of HMGB1 production, where HMGB1 levels peaked at days 8 to 10 p.i., and declined by day 14 p.i. (Fig. 2A). These data indicate that human influenza A isolates induce strong release of HMGB1 protein into circulation, although the temporal pattern of release varies depending on the particular isolate.

FluMist quadrivalent vaccine is a live attenuated vaccine that is administered nasally for active immunization of persons 2 to 49 years of age for the prevention of influenza disease caused by influenza A subtype and type B viruses (8). Although FluMist quadrivalent vaccine was recently discontinued, it was important to determine whether serum HMGB1 would also be induced upon FluMist inoculation as an indication of vaccine attenuation and safety. FluMist vaccine (2015–2016 formulation) was diluted 1:10 or 1:100 in phosphate-buffered saline (PBS) and was administered i.n. to young cotton rats. The doses used sought to approximate the equivalent of the human vaccination dose and to determine whether this attenuated vaccine would induce a dose-dependent response. We collected serum samples on 0, 2, 4, 7, 10, and 14 days after inoculation with FluMist and measured serum HMGB1 levels (Fig. 2B). Interestingly, rats treated with both doses of FluMist showed a late and much reduced increase in serum HMGB1 concentration compared to that seen during live influenza infection, evident at day 7 postvaccination, reaching peak concentration on day 10, and decreasing to basal levels by day 14 post-inoculation (Fig. 2B). Thus, this attenuated vaccine elicited a much lower level of HMGB1 than seen with live influenza A and B viruses.

### Comparison of serum HMGB1 responses in cotton rats upon infection by other respiratory viruses.

**FIG 3:**
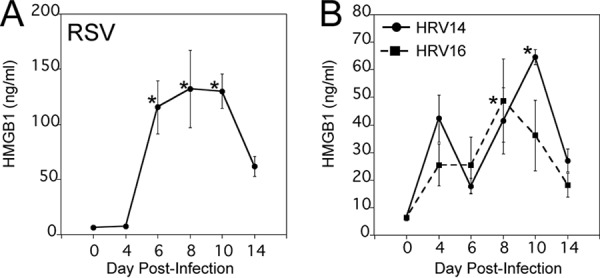
Kinetics of serum HMGB1 levels upon infection by other respiratory viruses in cotton rats. Cotton rats were infected i.n. with either 1 × 10^5^ PFU of RSV A/Long (A) or 1 × 10^7^ TCID_50_ of HRV14 or HRV16 (B). The levels of HMGB1 in serum samples were analyzed on the indicated days p.i. There were five cotton rats in each group. Values that were significantly different (*P* < 0.05) from the day 0 HMGB1 value are indicated by an asterisk.

In addition to influenza virus, cotton rats have been extensively used as a model to study respiratory syncytial virus (RSV) infection, as they are highly permissive for RSV with extended replication in nose and lung tissues (1 to 4 days p.i.) and a notable increase in lung cytokines and pathology (4 to 6 days p.i.) (30). Furthermore, we recently reported that cotton rats are semipermissive for replication of major human rhinovirus (HRV) groups, i.e., HRV14 and HRV16. These HRV serotypes exhibit a rapid replication cycle (1 to 2 days p.i.) in nose and lung tissues and elicit a mild increase in lung pathology (1 to 3 days p.i.) (31, 32). Animals were infected i.n. with RSV A/Long strain at 1 × 10^5^ PFU/cotton rat and, in the case of HRVs, with ~1 × 10^7^ TCID_50_/cotton rat, which evokes good replication and immune responses in cotton rats (30–33). Sera were collected at 0, 4, 6, 8, 10 and 14 days p.i. Similar to influenza virus infection, RSV A/Long infection of cotton rats induced a strong serum HMGB1 response; however, it was delayed in that it remained unchanged until day 4 p.i., was strongly increased by days 6 to 10 p.i., and declined by day 14 p.i. (Fig. 3A). Compared to RSV and influenza virus, both HRV14 and HRV16 induced lower peak levels of serum HMGB1 (Fig. 3B). The response to HRV14 was biphasic, with an early peak at day 4 p.i. and then a second peak at day 10 p.i., whereas the response to HRV16 exhibited a single peak. Overall, the release of HMGB1 protein into serum upon infection with RSV and HRVs mimic their strength of infection and reported inflammatory and pathology profile in cotton rats (30–32).

### Eritoran therapy prevents influenza B virus-induced serum HMGB1 levels *in vivo* and improves lung pathology without decreasing virus replication.

**FIG 4:**
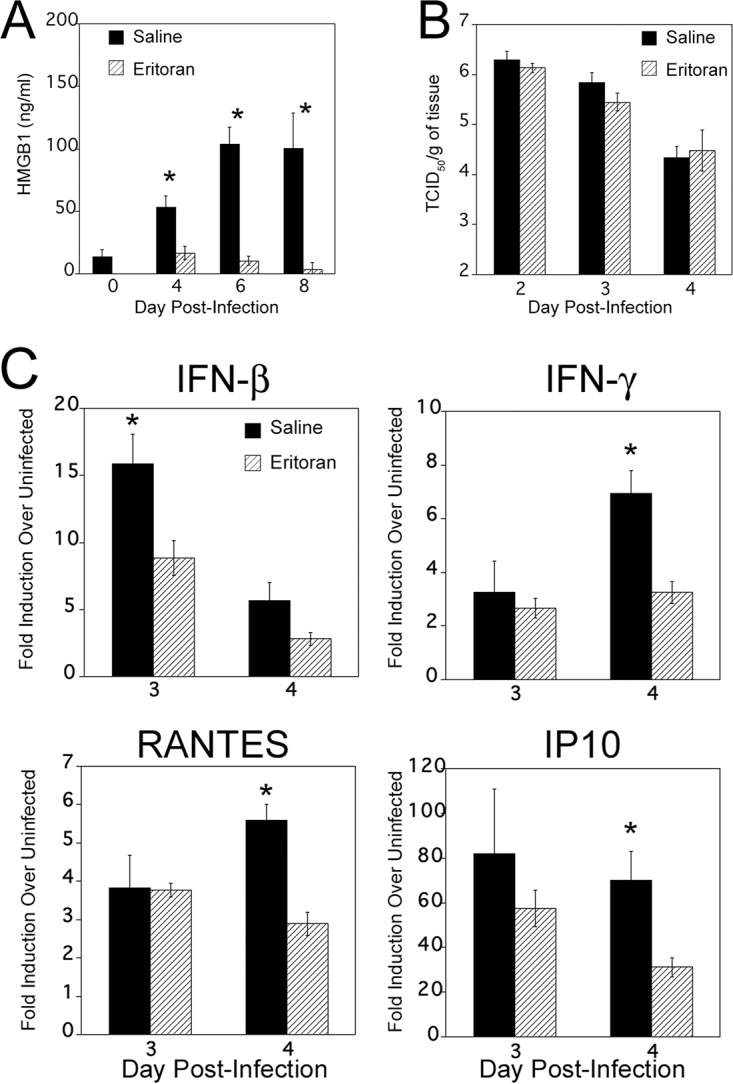
Eritoran treatment blocks influenza B virus-induced serum HMGB1 release *in vivo*. Cotton rats were infected i.n. with 1 × 10^6^ TCID_50_ of influenza B virus on day 0. On day 2 p.i., animals were treated with either 200 μl of saline (mock) or 37.3 mg/kg of Eritoran given by the retro-orbital (R.O.) route once daily until day 6 p.i. (A) Serum samples were collected on the indicated days p.i., and HMGB1 levels were measured. Results are from two independent experiments. The numbers of animals per treatment group were 10 (day 0), 20 (4 days p.i.), 15 (6 days p.i.), and 10 (8 days p.i.). Values for saline-treated animals that were significantly different (*P* less than 0.05) from the value for day 0 or Eritoran-treated HMGB1 are indicated by an asterisk. (B) Eritoran treatment does not affect virus replication *in vivo*. Animals were infected and treated as described above for panel A. On days 2, 3, and 4 p.i., animals from both saline- and Eritoran-treated groups were sacrificed 4 h after treatment, and nose tissues were collected for virus titration. There were five rats in each group. (C) Relative gene expression profile of IFN-β, IFN-γ, IP10, and RANTES in the lung tissues of saline- or Eritoran-treated, influenza B virus-infected cotton rats. Groups of cotton rats (five animals in each group) were euthanized on the indicated day p.i., and the lungs were collected for cytokine mRNA analysis by qRT-PCR. The results were calculated as fold induction for each cytokine over the level of expression in uninfected animals and expressed as geometric means ± SEMs. Values for saline-treated animals that are significantly different (*P* < 0.05) from the values for Eritoran-treated animals are indicated by an asterisk.

Previously, we showed that cotton rats infected with a nonadapted human influenza A California pH1N1 virus induced strong serum HMGB1 release, which was inhibited by Eritoran treatment *in vivo* (14). To investigate whether Eritoran treatment also blocked influenza B virus-induced serum HMGB1 levels, cotton rats were infected i.n. with 1 × 10^6^ TCID_50_ of influenza B virus, and starting at day 2 p.i., animals were treated once daily with either saline or Eritoran through day 6 p.i. Eritoran treatment significantly reduced influenza B virus-induced HMGB1 levels on days 4, 6, and 8 p.i. to basal levels (Fig. 4A). As influenza virus is cleared quickly from the cotton rat lung and is normally not detected on day 2 p.i., the time when Eritoran treatment was initiated, we evaluated the effect of Eritoran on influenza virus replication in the cotton rat nose, where the virus remains replicating longer that in the lung (27). Figure 4B shows that there was no difference in viral loads in nasal tissues at days 2, 3, and 4 p.i. between saline and Eritoran treatment, indicating that Eritoran treatment administered therapeutically does not affect virus replication in the upper respiratory tract, despite its ability to blunt serum HMGB1 levels.

In cotton rats, influenza virus infection rapidly induces many inflammatory cytokines starting soon after infection (within 1 to 3 days p.i.), including beta interferon (IFN-β), interleukin 6 (IL-6), and tumor necrosis factor alpha (TNF-α), coinciding with virus replication, and these cytokine levels wane by days 6 to 8 p.i. (27). Thus, we measured expression of select cytokines in the lung after infection with influenza B virus and treatment with either saline or Eritoran (i.e., IFN-β, IFN-γ, RANTES, and IP10) (Fig. 4C). Compared to saline-treated cotton rats, Eritoran treatment resulted in significantly lowered mRNA expression of these four cytokines in the lung, with the highest impact occurring at 4 days p.i., with an early effect on day 3 for IFN-β.

**FIG 5:**
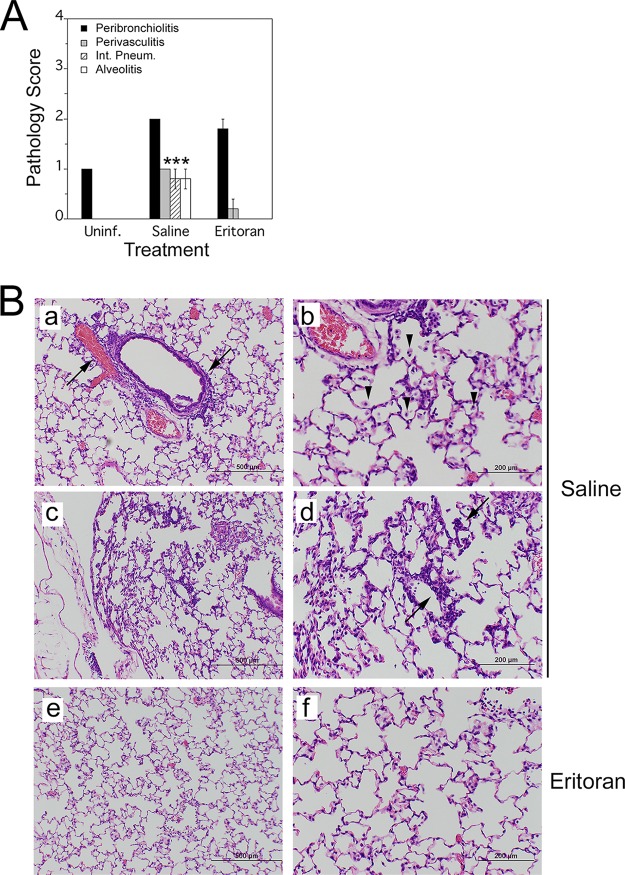
Eritoran attenuates late influenza B virus-induced lung pathology. (A) Histopathology scores (extent of peribronchiolitis, perivasculitis, interstitial inflammation, and alveolitis) were assessed in the lungs of uninfected or influenza B virus-infected cotton rats treated with either saline or Eritoran, euthanized at 8 days p.i. There were five rats in each group. Values that are significantly different (*P* < 0.05) for the saline versus Eritoran treatment are indicated by an asterisk. (B) Representative H&E-stained sections of lungs from rats treated with saline (a, b, c, and d) or Eritoran (e and f) on day 8 p.i. Panels a and b show increased peribronchiolitis and perivasculitis (black arrows) in addition to marked alveolar mononuclear cell infiltration (black arrowheads) in animals treated with saline. Panels c and d show unresolved interstitial pneumonia in saline-treated animals (black arrow). Panels e and f show resolution of interstitial pneumonia in Eritoran-treated animals. Magnifications, ×40 (a, c, and e) and ×100 (b, d, and f).

We subsequently determined the extent of lung pathology in cotton rats that were infected with influenza B virus and treated with either saline or Eritoran by scoring hematoxylin-and-eosin (H&E)-stained lung sections at 8 days p.i. Influenza B virus infection significantly increased peribronchiolitis, perivasculitis, interstitial pneumonia, and alveolitis at 8 days p.i. (Fig. 5A, compare saline treatment versus uninfected). Importantly, Eritoran treatment significantly reduced the extent of perivasculitis, interstitial pneumonia, and alveolitis (Fig. 5A, compare saline versus Eritoran treatment; Fig. 5B, compare panels a, b, c, and d to panels e and f), indicating that Eritoran treatment was also efficacious at reducing lung pathology induced by influenza B infection.

## DISCUSSION

Influenza continues to be a serious public health problem that affects people in all age groups and causes severe illness and death in high-risk populations. Influenza epidemics cost billions of dollars annually (2, 4). Seasonal influenza vaccination is the most effective way to prevent disease; however, precisely predicting the annually mutating immunogenic epitopes, as well as production and distribution issues, have taken an economic toll (9). The emergence of influenza viruses that are resistant to antivirals has also created a critical demand to develop new, safe, and effective alternative therapeutics (10).

To date, immunologic research has focused mostly on foreign antigens as a source of immune recognition. However, it is now increasingly clear that along with well-established pathogen-associated molecular patterns (PAMPs), endogenous DAMPs also drive immune responses and that DAMP release may serve as a marker of disease progression and/or severity or dictate possible complications at later stages of infection (34). In line with these ideas, DAMP involvement has been firmly established in many inflammatory and infectious diseases (34). One of the best-known examples is HMGB1, first studied for its role as a DNA-binding protein facilitating gene transcription. However, extracellular HMGB1 is now recognized as a critical late mediator of sepsis (17, 18). HMGB1 is actively released from stimulated macrophages and monocytes and passively released from necrotic but not apoptotic cells, and functions as either a chemokine facilitating movement of immune cells to sites of infection or further activates other immune cells to secrete proinflammatory cytokines, thus aggravating the inflammatory response (16).

In the case of influenza virus infection in a murine model, circulating levels of HMGB1 in plasma peaked relatively late on day 9 p.i. by A/Japan/305/57 (H2N2), a time that did not correlate with peak mortality in mice (day 7 p.i.). Moreover, treatment of mice with ethyl pyruvate to reduce secretion of HMGB1 did not reduce mortality; thus, a role for HMGB1 release in influenza virus infection was not clearly established in this report (19). However, anti-HMGB1 monoclonal antibodies significantly improved the survival rate of H1N1-infected mice, without affecting virus replication in the lungs, and significantly attenuated influenza virus-induced histological changes, neutrophil infiltration in the lungs, and inflammatory cytokine/chemokine expression (35). Furthermore, serum HMGB1 levels were found to be significantly higher in patients with 2009 pandemic H1N1 influenza virus-associated encephalopathy with poor outcomes compared to patients without neurological complications, and a positive correlation between HMGB1 and IL-6 levels in serum was found in these patients (36). Since the temporal changes in systemic HMGB1 levels during the course of infection with various strains of influenza virus had not been previously examined, we sought to establish whether an association of HMGB1 release with influenza virus infection progression existed and, if it did, whether this information could be used to define subsequent therapies. To this end, cotton rats were infected with nonadapted strains of influenza A and B viruses, since viral adaptation introduces variations that may dramatically alter the course of natural infection (23, 24). In addition, the course of influenza virus infection and associated histopathological changes seen in cotton rats are more relevant to human infection and disease than in the mouse model (27). Importantly, cotton rats are permissive to infections with most of the common human viral respiratory pathogens, including RSV, HRVs, human metapneumovirus, human parainfluenza virus, adenoviruses, enterovirus D68, and measles virus, making this species an excellent model for studying human respiratory viruses (30–32, 37, 38).

During seasonal influenza epidemics, similar clinical features have been seen in patients infected with influenza A and B viruses in outpatient settings (39). Substantial influenza B virus infections were noted among pediatric influenza-associated fatalities (40), and it has been suggested that oseltamivir may be less effective at reducing fever in outpatients infected with influenza B virus compared to influenza A virus (41). In addition, a study published by CDC and partners showed that among hospitalized adults, influenza B viruses caused equally severe disease outcomes and clinical characteristics, including length of stay, ICU admission, and death, as influenza A viruses, suggesting influenza B virus infections can be just as severe as influenza A virus infections (5). Therefore, we included both influenza type A and B viruses in our study and measured serum HMGB1 levels systematically before and after infection. Both influenza A and B virus infections induced robust levels of serum HMGB1 levels in a time-dependent manner, although induction profiles differed somewhat among the strains (Fig. 1A and 2A). Overall, serum HMGB1 increased significantly by days 4 to 6 p.i., peaked from days 6 to 10 p.i. (depending on the infecting strain), and slowly returned to basal levels by days 14 to 18 p.i. These temporal differences in serum HMGB1 release among different strains could represent differences in replication potential or severity of the disease, which these strains cause in cotton rats. The absence of induction of serum HMGB1 with cotton rats inoculated with UV-inactivated influenza B virus and a dose-dependent effect on HMGB1 levels suggest that this response is likely to be dependent on replication (Fig. 1A and C). Interestingly, the expression of type I IFN-regulated Mx proteins in the lungs of RSV- and influenza virus-infected cotton rats was defined previously as a marker of local inflammation, typically peaking on day 7 p.i. for influenza virus and on days 4 to 6 p.i. for RSV (42, 43). Together, our data indicate that systemic HMGB1 release during both RSV and influenza virus infection in cotton rats correlates with the robustness of the inflammatory response assessed by type I IFN response in the lungs.

Cold-adapted, live attenuated, temperature-sensitive FluMist vaccine replicates in the lining of the nasopharynx for up to 28 days after administration to induce immunity, and it confers protection by stimulating production of mucosal IgA, systemic IgG, and T cells (8). Thus, FluMist mimics aspects of the infectious process, such as the initial invasion of host cells, but fails to infect host cells beyond the nasopharynx. FluMist also induced significant serum HMGB1 release (Fig. 2B), although the serum HMGB1 concentration was lower than the live influenza virus strains tested (compare the *y* axes of Fig. 1A and 2A with Fig. 2B). As live-virus preparations and FluMist differ in their total virus inocula, 2 dilutions of the attenuated strain were used to approximate the dose used in humans. It is possible that moderate HMGB1 release upon FluMist inoculation is required to induce an optimal protective response without compromising safety. In this regard, the use of HMGB1 as an adjuvant during both HIV antigen (Ag)-encoded and influenza virus Ag-encoded DNA vaccination was proposed to modulate inflammation by stimulating the recruitment and activation of dendritic cells to sites of vaccine-encoded Ag production (44, 45).

Our data further show that infection with RSV, HRV14, and HRV16 also causes temporally defined release of serum HMGB1; however, the strength of HMGB1 release in RSV-infected animals was stronger than that in HRV14- and HRV16-infected animals and comparable to animals infected with influenza A and B viruses (Fig. 3A and B). Overall, our data indicate that the kinetics of induction of serum HMGB1 parallels the length of time of the replication cycle and relative levels correlate with severity of infection by these viruses in cotton rats as defined by the extent of lung cytokine expression and pathology (30–33). Our data are in agreement with previous findings describing differences in the intensities of the whole-blood gene expression profiles studied during influenza virus, RSV, and HRV infections in a pediatric population (46). Along with apoptosis, viruses can trigger an alternative form of caspase-independent cell death called programmed necrosis (or necroptosis), which is emerging as an additional host defense mechanism that restricts virus persistence in cases when apoptosis is inhibited by the virus (47). It has been shown that influenza virus induces necroptosis in lung epithelial cells and that HMGB1 is released from necroptotic cells (48, 49). It is not clear how HMGB1 release is caused during HRV infections, but interestingly, a recent report has shown that RSV infection of human airway epithelial cells induced a significant HMGB1 release into the extracellular space that can act as a paracrine factor that primes epithelial cells and monocytes to inflammatory stimuli in the airways (50). Thus, it is possible that depending on the duration and severity of the replication cycle of the various viruses, differing numbers of necroptotic cells cause variations in the duration or peak levels of systemic HMGB1 release.

In the case of influenza and other microbial infections, therapeutic approaches that interfere with either virus- or DAMP-induced TLR4 signaling may open up new possibilities for treatment against pathogenesis (11–14, 21). Cumulatively, our mouse and cotton rat data with Eritoran, a TLR4 antagonist developed for the treatment of sepsis, showed that Eritoran treatment inhibited influenza virus-induced, (i) oxidized phospholipid expression-mediated cytokine storm *in vivo* and *in vitro*, (ii) HMGB1-mediated TLR4-dependent signaling *in vitro*, and (iii) HMGB1 release into serum *in vivo*, and improved influenza-induced lethality, lung pathology, and clinical symptoms (13, 14). Furthermore, a small-molecule inhibitor of HMGB1, P5779, shown previously to protect mice against hepatic ischemia/reperfusion injury and sepsis, also significantly protected mice against influenza-induced lethality and lowered clinical scores, comparable to Eritoran treatment (14, 15). As influenza B virus infections are as severe as influenza A virus infections, the data presented herein extend our previous findings by demonstrating that Eritoran treatment also significantly blocked nonadapted influenza B virus-induced systemic HMGB1 release *in vivo* and improved lung pathology (Fig. 4A and Fig. 5A and B). We did not observe any change in nose viral titers in the cotton rats upon Eritoran treatment compared to saline treatment (Fig. 4B). This is an important observation because it indicates that it is influenza virus-induced pathology, and not the viral titers *per se*, that causes ALI and supports our previous findings for mice infected with a mouse-adapted influenza A virus strain (13).

Eritoran is not a specific HMGB1 inhibitor; rather, it is an analog of lipopolysaccharide (LPS) and occupies a deep hydrophobic pocket in MD-2 (extracellular TLR4 adapter) and competitively inhibits binding of the lipid A region of LPS to the same pocket and blocks dimerization of TLR4 (51). We also showed that Eritoran fails to protect CD14^−/−^ mice from PR8 infection, as well as inhibits transfer of LPS from CD14 (coreceptor of LPS) to MD-2, indicating that Eritoran likely binds to CD14 as well (13). Since HMGB1-induced TLR4 signaling has been found to be dependent on both MD-2 and CD14 (15, 20), Eritoran likely inhibits the interaction of HMGB1 with these two coreceptors as well. In contrast, the small-molecule HMGB1 inhibitor P5779 binds outside the deep hydrophobic pocket of MD-2 and blocks HMGB1-induced TLR4 signaling, but not LPS-induced signaling (15). At this time, we do not know how Eritoran blocks release of HMGB1 into serum after virus infection; however, we hypothesize that epithelial necroptosis caused by influenza virus infection (49) leads to release of HMGB1 that, in turn, engages TLR4 and can be blocked by both Eritoran and P5779.

HMGB1 expresses three cysteine residues (C23, C45, and C106) and their redox states determine the biological function of HMGB1. Distinct isoforms of HMGB1 have different activities. Fully reduced HMGB1, which expresses three cysteine thiol residues, exerts chemotactic activity (16). Mildly oxidized HMGB1, where C23 and C45 form a disulfide link and C106 possesses a thiol residue, has proinflammatory and cytokine-stimulating activity (16). HMGB1 with any of the cysteines terminally oxidized (sulfonyl) has no identified immune-modulating activity (16). While we are unable to determine which isoforms are being elicited by virus infection due to a lack of isoform-specific antibodies, the fact that blocking TLR4 signaling with Eritoran inhibits HMGB1 release into serum in virus-infected mice and cotton rats and the fact that the HMGB1 antagonist P5779 also binds to the TLR4 MD-2 coreceptor would suggest that it is the disulfide isoform that is active in our system (14, 15). Future experiments will be required to confirm this conclusion.

Taken together, our data indicate that HMGB1 release seems to be a common outcome, irrespective of whether influenza A or B virus was tested. Thus, therapies that block HMGB1 release or action, such as Eritoran or P5779, should be considered universal, adjunctive therapies to influenza vaccines.

## MATERIALS AND METHODS

### Animals.

Animal experiments were conducted in accordance with the recommendations in the *Guide for the Care and Use of Laboratory Animals* (52). The animal protocols were approved by the Institutional Animal Care and Use Committee (IACUC) of Sigmovir Biosystems, Inc. (SBI) (Office of Laboratory Animal Welfare [OLAW] assurance A4642-01). Four- to six-week-old, male or female cotton rats were obtained from the inbred colony maintained at SBI. Animals were housed in large polycarbonate cages and fed a diet of standard rodent chow and water *ad libitum*. The animals were seronegative for adventitious respiratory viruses and other common rodent pathogens. All infections were strictly carried out at standard biosafety level 2. Cotton rats were infected i.n. with 100 μl of live influenza A or B virus, RSV, or HRVs (stock titers of each virus presented below) or inoculated with 100 μl of UV-inactivated influenza B virus or with FluMist (MedImmune, Gaithersburg, MD) under isoflurane anesthesia as indicated in the figure legends. Serum samples were obtained by R.O. blood collection under isoflurane anesthesia. Animals were euthanized by carbon dioxide asphyxiation.

### Viruses.

The preparation and source of influenza A/California 04/2009 virus (stock titer, 4.3 × 10^7^ TCID_50_/ml) and A/Wuhan/359/95 virus (stock titer, 1 × 10^7^ TCID_50_/ml) were previously described (23, 27). A/Victoria H3N2 (stock titer, 6.8 × 10^6^ TCID_50_/ml) virus was obtained by harvesting the supernatants of MDCK cells that had been inoculated 3 days previously at a low multiplicity of infection (MOI). Influenza B/Wisconsin virus (stock titer, 5 × 10^7^ TCID_50_/ml) was propagated in 10-day-old, pathogen-free, embryonated chicken eggs. The titers of all virus stocks were determined by standard endpoint dilution assays on MDCK cells as described below. MDCK cells were maintained in minimal essential medium containing Earle salts (EMEM) supplemented with 10% fetal bovine serum (FBS). Inactivated preparations of B/Wisconsin virus were generated by exposing the virus stocks to UV irradiation for 45 min at 100 mJ/cm^2^ and confirmed by virus titration assay.

The prototype Long strain of RSV (RSV A/Long) (ATCC catalog no. VR-26) (stock titer, 5 × 10^7^ PFU/ml) was propagated in HEp-2 cells, and its infectious titer was determined as previously described (33). HRV14 (ATCC catalog no. VR-284) (stock titer, 8.62 × 10^8^ TCID_50_/ml) and HRV16 (ATCC catalog no. VR-283) (stock titer, 4 × 10^8^ TCID_50_/ml) were produced in HeLa Ohio (HeLa OH) cells as previously described (32), and stock titers were determined by virus titration assay described below.

### Virus titration assay.

For influenza viruses, confluent monolayers of MDCK cultures growing in 96-well plates were infected with serial 10-fold dilutions of virus stock preparations, lung or nose homogenates (each dilution was tested in quadruplicate) in the presence of 1 μg/ml L-1-tosylamide-2-phenylethyl chloromethyl ketone (TPCK)-treated trypsin. After the cells were incubated for 5 days at 37°C, cells were stained with crystal violet, and cytopathic effect (CPE) was scored, and virus titer was expressed as TCID_50_ per milliliter by the method of Reed and Muench (53).

For HRVs, confluent monolayers of H1-HeLa cells in 96-well plates were infected with serial 10-fold diluted virus stock preparation in quadruplicate and incubated at 33°C for 5 days, followed by staining with crystal violet and scoring the plates for CPE to express the titers as TCID_50_/ml as described above.

### Virus challenge followed by treatment with Eritoran.

Cotton rats were i.n. infected with influenza B/Wisconsin (1 × 10^6^ TCID_50_/100 μl/rat). Two days after infection, cotton rats received either vehicle (saline) or Eritoran (37.3 mg/kg of body weight per rat in 200 μl) through the R.O. route once daily (days 2 to 6).

### HMGB1 ELISA.

Blood samples were collected on the indicated days p.i. by the R.O. route. Serum samples were processed within 1 h of blood collection by centrifuging the tubes at 3,000 rpm for 10 min at 4°C, and sera were stored in a −20°C freezer. Serum HMGB1 levels were measured using a commercially available ELISA kit according to the manufacturer’s instructions (IBL International, Canada).

### RNA isolation and quantitative reverse transcription-PCR (qRT-PCR) analysis.

Briefly, RNA was isolated from the lung lingular lobe using the RNeasy kit (Qiagen Sciences) and cDNA was prepared by QuantiTect reverse transcription kit (Qiagen Sciences) according to the manufacturer’s instructions. Each cDNA reaction was prepared from 1 μg of RNA, diluted to 100 μl of the final volume, and 3 μl of cDNA was subsequently used for each PCR. The assessment of cotton rat-specific cytokine mRNA expression was carried out using primers as previously described (38). The level of each cytokine mRNA was normalized by comparison to expression of the β-actin housekeeping gene and reported as fold induction over the level in uninfected lungs.

### Lung histopathology.

The lungs (right lobe) were dissected, inflated with 10% neutral buffered formalin, and immersed in formalin for fixation. The lungs were embedded in paraffin blocks, sectioned, and stained with H&E. The slides were read in a blind manner and examined for four parameters of pulmonary inflammation, i.e., peribronchiolitis, perivasculitis, interstitial pneumonia, and alveolitis, as previously described (31).

### Statistical analysis.

Serum HMGB1 levels were calculated as geometric means ± SEMs for all animals in a group at a given time p.i. Statistical difference between the values for two groups was determined using an unpaired, two-tailed Student’s *t* test, whereas more than two groups were analyzed by one-way analysis of variance (ANOVA) with Bonferroni *posthoc* test; *P* < 0.05 was chosen as the level of significance for both analyses.
